# *Streptococcus pneumoniae* meningitis in a child with idiopathic nephrotic syndrome: a case report

**DOI:** 10.1186/s13256-022-03648-5

**Published:** 2022-11-07

**Authors:** David Guernsey, Aparna Arun, Rabia Agha, Juan C. Kupferman

**Affiliations:** Department of Pediatrics, Maimonides Medical Center, 977 48th Street, Brooklyn, NY 11219 USA

**Keywords:** Infection, Central nervous system, Kidney, Pediatric, Meningeal

## Abstract

**Background:**

Children with nephrotic syndrome are at increased risk of infections, including bacterial peritonitis, pneumonia, and cellulitis. However, bacterial meningitis, a potentially life-threatening complication, has not been highlighted as an infectious complication of nephrotic syndrome in recent reviews. We report a very subtle and unusual presentation of bacterial meningitis in a child with nephrotic syndrome, which without a high index of suspicion, would have been missed.

**Case presentation:**

A 9-year-old African-American male with a history of steroid-dependent nephrotic syndrome presented to the nephrology clinic for routine follow-up. His medications included mycophenolate mofetil and alternate-day steroids. His only complaint was neck pain and stiffness that the mother attributed to muscle tightness relieved by massage. There was no history of fever, vomiting, headache, photophobia, or altered mental status. On physical examination, he was afebrile (99 °F), but had mild periorbital swelling and edema on lower extremities. He appeared ill and exhibited neck rigidity, and demonstrated reflex knee flexion when the neck was bent. Laboratory evaluation revealed leukocytosis, elevated C-reactive protein, hypoalbuminemia, and proteinuria. Cerebrospinal fluid suggested bacterial meningitis. The patient was treated with ceftriaxone and vancomycin. Both cerebrospinal and blood cultures grew *Streptococcus pneumoniae*; vancomycin was discontinued. The child completed a 2-week course of ceftriaxone and was discharged home.

**Conclusions:**

A high index of suspicion is necessary in children with nephrotic syndrome treated with corticosteroids, as symptoms may be masked, and thus, a life-threatening disease be missed. Bacterial meningitis should be highlighted as a serious infection complication in children with nephrotic syndrome.

## Introduction

Infections are leading causes of morbidity in children with nephrotic syndrome (NS). Children with NS are at increased risk of bacterial peritonitis, pneumonia, and cellulitis, as well as sinusitis/tonsillitis, gastroenteritis, and urinary tract infections [1, 2]. Bacterial meningitis, a serious complication of NS, has not been highlighted as a potential infection in NS in recently published reviews, despite the growing number of case reports of this serious complication [3–6].

Here we present a subtle case of *Streptococcus pneumoniae* meningitis in a child with NS who presented without fever and with minimal symptoms, and emphasize the need to have a high index of suspicion in children treated with corticosteroids as symptoms may be masked and a life-threatening disease missed.

## Case presentation

A 9-year-old African-American male with a history of steroid-dependent NS presented to the nephrology clinic for routine follow-up care in September 2018. He had been diagnosed with NS at 4 years of age, and had multiple relapses and hospitalizations for management of his disease. He was up-to-date with all his immunizations, including vaccination for *S. pneumoniae;* he had received PCV7 as the initial series and a booster of PCV13. He was receiving mycophenolate mofetil 200 mg twice a day for about 6 months and prednisone 30 mg on alternate days for about 1 month. The child’s only complaint was neck pain and stiffness that had begun 1–2 days prior to presentation. His mother attributed the neck pain to muscle tightness and stated that massages to the area provided some pain relief. There was no history of recent illness, fever, nausea, vomiting, photophobia, headache, seizures, or altered mental status.

On physical examination, he was afebrile and had mild periorbital swelling and edema on lower extremities. He appeared ill, exhibited neck rigidity, and revealed reflex knee flexion on neck flexion. He was transferred to the emergency department (ED) for further evaluation and management. On admission to the ED, his temperature was 99 °F, heart rate 112 beats per minute, respiratory rate 22 breaths per minute, blood pressure 101/72 mmHg, and oxygen saturation 99% on room air. The child persisted with neck stiffness, and roughly 7 hours after presenting to the ED, he developed fever (102 °F). At this time, a lumbar puncture was performed to rule out meningitis. Laboratory evaluation showed mild anemia, leukocytosis with neutrophil predominance, hyponatremia, elevated C-reactive protein (CRP), and an elevated sedimentary rate. Cerebrospinal results demonstrated pleocytosis, with very decreased glucose level. Laboratory findings are summarized in Table 1.

**Table 1:** Laboratory evaluation

	Result	Normal range	Units
General hematology			
WBC	18.4	4.5–13.0	K/µL
Neutrophil count	86	50–60	%
Bands	7	0–8	%
HGB	10.6	11.5–15.5	g/dL
HCT	32.4	35.0–45.0	%
Platelets	160	150–400	K/µL
General chemistry			
Sodium, serum	128	135–149	mmol/L
Potassium, serum	3.9	3.4–4.8	mmol/L
Chloride, serum	100	93–105	mmol/L
Carbon dioxide, serum	22	23–32	mmol/L
Glucose, serum	113	59–140	mg/dL
BUN, serum	19	7–21	mg/dL
Creatinine, serum	0.5	0.3–0.7	mg/dL
Calcium, serum	6.4	8.7–10.0	mg/dL
Magnesium	2.3	1.6–2.2	mg/dL
Phosphorus	3.8	2.2–5.5	mg/dL
Albumin, serum	< 1.5	3.5–5.2	g/dL
Sedimentation rate	110	0–15	mm/hour
C-reactive protein	16.38	0.0–0.9	mg/dL
Total protein, CSF	179	20–100	mg/dL
Glucose, CSF	< 10	40–170	mg/dL
CSF WBC	110	0–10	Per µL
CSF RBC	57		Per µL
CSF neutrophil	40	0–6	%
CSF lymphocyte	56	40–80	%
CSF monocyte	4	15–45	%
Urine protein	300	Negative	mg/dL
Urine hemoglobin	Small	Negative	
Urine WBC	5–10	< 5	Per HPF
Urine RBC	5–10	0	Per HPF

*WBC* white blood cells, *HGB* hemoglobin, *HCT* hematocrit, *BUN* blood urea nitrogen, *CSF* cerebrospinal fluid, *RBC* red blood cells, *K/µL* thousands per microliter, *g/dL* grams per deciliter, *mmol/L* millimoles per liter, *mg/dL* milligrams per deciliter, *mm/hour* millimeters per hour, *µL* microliter, *HPF* high-power field

The patient was started on ceftriaxone 100 mg/kg/day and vancomycin 15 mg/kg/day, and was admitted to the pediatric intensive care unit (PICU) for close cardiopulmonary monitoring and further management.

By the second day of hospitalization, both cerebrospinal and blood cultures grew *S. pneumoniae,*, which was pansensitive, including to ceftriaxone. Vancomycin was discontinued and the child remained on ceftriaxone monotherapy at 100 mg/kg. He was restarted on daily steroid therapy to attain remission of the NS. While in the PICU, the patient developed persistent hypertension and was treated with enalapril 5 mg twice a day. Amlodipine 5 mg once a day was added later as a second antihypertensive to further optimize blood pressure (BP) control. The child was transferred to the inpatient ward where he completed a 2-week course of ceftriaxone with no complications. His overall edema improved, and his BP was controlled on two antihypertensive drugs. He was discharged home on prednisolone, enalapril, and amlodipine, with close follow-up with nephrology as an outpatient.

## Discussion

NS is characterized by the presence of proteinuria, hypoalbuminemia, edema, and hyperlipidemia [1, 7]. Minimal change disease and focal segmental glomerulosclerosis are the most common causes of NS in the pediatric population [8]. Regardless of the inciting event or causes, the pathophysiology of the disease remains the same: a dysfunction of the glomerular filtration barrier leading to loss of protein in the urine.

The initial treatment for idiopathic minimal change disease typically involves an 8–12-week course of corticosteroids. About 80% of patient will achieve remission; those who respond are classified as steroid sensitive, and those who do not, are steroid resistant [9]. While the majority will achieve remission, many patients will later develop relapses for various causes, including infections, stress, or environmental factors. Corticosteroids are still the first line of treatment for the relapses, leading to long courses of treatment. Other medications such as mycophenolate mofetil, cyclophosphamide, cyclosporine, or rituximab are utilized in cases of steroid dependence or steroid resistance to decrease the use of steroids or to achieve remission, respectively [7].

Infection remains the leading cause of mortality for children with NS. These children are at increased risk of bacterial peritonitis, pneumonia, and cellulitis; other reported infections include sinusitis/tonsillitis, gastroenteritis, and urinary tract infection [1, 2]. Children with NS have compromised immune systems due to the disease as well as its treatment. In addition to increased loss of urinary albumin, immunoglobulin G (IgG) is lost in the urine of nephrotic children as well [1]. As a result, these children are effectively hypogammaglobulinemic and, thus, have a reduced capacity to fight bacterial infections, especially with encapsulated organisms such as *S. pneumoniae*, as these encapsulated organisms require IgG for opsonization. In addition to *S. pneumoniae*, these patients are at increased risk of infection with *Neisseria meningitidis* and *Salmonella* species, as well as Enterobacteriaceae. Proteins of the complement system, which play a critical role in the adaptive and innate immune system, may also be excreted in excess in the urine [10]. As stated before, glucocorticoids remain the mainstay of treatment that often require multiple and prolonged courses; steroid therapy increases infection risk [11]. Other immunosuppressive agents, such as mycophenolate mofetil and calcineurin inhibitors (that is cyclosporin and tacrolimus), have also been shown to increase the risk of infection [12].

Bacterial meningitis as a complication of NS has not been highlighted as a potential infection in NS in recent reviews [1, 2]. A recent study that examined pneumococcal sepsis in patients with NS in India over a 14-year period, found only five cases of bacterial meningitis [5]. Typically, bacterial meningitis presents with the sudden onset of fever, headache, and neck stiffness that is usually accompanied by nausea, vomiting, photophobia, and altered mental status. Other reported cases of bacterial meningitis in NS presented with fever, nausea or vomiting, headaches, seizures, or altered mental status, as presenting symptoms [3, 4, 6]. These patients, even without the diagnosis of NS, would have warranted an evaluation for potential meningitis. In contrast, our patient had a subtle clinical presentation without fever, headache, photophobia, or altered mental status. Thus, our case highlights the potential subtle meningitis presentation in patients with NS, which without a detailed evaluation would be missed. The consequences of missing severe bacterial meningitis are very serious, including hearing loss, brain damage, and in rare cases, death.

Previous reported cases of bacterial meningitis in children with NS have occurred in resource-poor countries with incomplete vaccination or with organisms not covered by routine childhood vaccines [3–6]. In contrast, our patient developed an invasive *S. pneumoniae* infection even in a country as resource rich as the USA, with universal access to childhood immunizations. It is, therefore, important to alert providers treating children with NS of this severe complication, regardless of country of origin and/or vaccination or socioeconomic status.

The incidence of bacterial meningitis has significantly declined since the 1980s after the introduction of the *Haemophilus influenzae* type b and *S. pneumoniae* vaccines [13]. Still, invasive pneumococcal disease remains the most common cause of bacterial meningitis [13, 14]. The first pneumococcal vaccine developed in 1983, the 23-valent pneumococcal polysaccharide vaccine (PPSV23), protected adults as well as children older than 2 years of age against invasive disease caused by the capsular serotypes contained in the vaccine. The seven-valent pneumococcal conjugate vaccine (PCV7) was developed in 2000 for use in children younger than 2 years of age. The latest vaccine to be developed in 2010, the 13-valent pneumococcal conjugate vaccine (PCV13), contains the seven serotypes in PCV7, five additional serotypes from PPSV23, and a new serotype not contained in PPSV23 or PCV7 [15].

According to the most recent Active Bacterial Core surveillance, a core component of the Center for Disease Control and Prevention’s Emerging Infections Programs networks, there were 225 cases of *S. pneumoniae*-related meningitis in the USA, despite the use of the PCV13 vaccine in 2019. The number has significantly decreased since the institution of the conjugate vaccines, but is still considerable, especially in the immunocompromised population [16]. Unfortunately, there are still considerable number of *S. pneumoniae* serotypes that are not covered by the current vaccines [17]. Because of the declining incidence rates, physicians must remain vigilant and consider the diagnosis of meningitis, especially in high-risk groups such as NS [18]. The diagnosis still requires a high index of suspicion to obtain the proper diagnostic tests and provide the appropriate treatment to achieve positive outcomes [19].

The mechanism by which the patient developed *S. pneumoniae* meningitis is not clear. The patient did have a history of spontaneous bacterial peritonitis from *S. pneumoniae*. The patient may have been colonized leading to bacteremia and then had an hematogenous spread into the cerebrospinal fluid (CSF). The serotype of the pneumococcus for this patient was not identified, limiting our ability to understand the impact of the patient’s vaccination status on his presentation. As mentioned before, the patient completed the pneumococcal vaccine series; however, he still developed pneumococcal meningitis. As noted above, the PCV13 series does not include all serotypes or all invasive serotypes, and our patient had not received the PPSV23. The lack of coverage for various serotypes may explain how a person vaccinated for pneumococcal disease could develop a severe, invasive infection.

## Conclusion

This case of meningitis illustrates a very serious complication of NS that not only was very subtle at presentation, but also rarely identified as a risk of NS. Classic symptoms may be masked owing to chronic steroid use. This elusive case of meningitis could have easily been overlooked and missed. The consequences of missing the diagnosis of meningitis are very severe. Thus, we believe that bacterial meningitis should be highlighted as a possible serious complication of NS.

## Data Availability

All data generated or analyzed during this study is available on the patient’s medical chart, which is not publicly available.
